# A Multivariate Time-Series Based Approach for Quality Modeling in Wireless Networks

**DOI:** 10.3390/s21062017

**Published:** 2021-03-12

**Authors:** Leonardo Aguayo, Sergio Fortes, Carlos Baena, Eduardo Baena, Raquel Barco

**Affiliations:** 1 Departamento de Engenharia Elétrica, Universidade de Brasília, Campus Universitário Darcy Ribeiro, Brasília-DF 70910-900, Brazil; aguayo@unb.br; 2 Departamento de Ingeniería de Comunicaciones, Andalucía Tech, Campus de Teatinos s/n, Universidad de Málaga, 29071 Málaga, Spain; jcbg@ic.uma.es (C.B.); ebm@ic.uma.es (E.B.); rbm@ic.uma.es (R.B.)

**Keywords:** mobile networks, modeling, KQI, QoE, machine-learning

## Abstract

This work presents a method for estimating key quality indicators (KQIs) from measurements gathered at the nodes of a wireless network. The procedure employs multivariate adaptive filtering and a clustering algorithm to produce a KQI time-series suitable for post-processing by the network management system. The framework design, aimed to be applied to 5G and 6G systems, can cope with a nonstationary environment, allow fast and online training, and provide flexibility for its implementation. The concept’s feasibility was evaluated using measurements collected from a live heterogeneous network, and initial results were compared to other linear regression techniques. Suggestions for modifications in the algorithms are also described, as well as directions for future research.

## 1. Introduction

Real-time service quality assessment is one of the critical aspects of modern wireless networks. However, its practical implementation faces some challenges. One of them is scalability: the sustained increase in the number of nodes demands fast, stable, and distributed data processing. IoT and M2M scenarios encountered in 5G and beyond systems put even more pressure on the monitoring systems to cope with the constant increment of traffic volume.

A second aspect is a need, from the service perspective, to measure quality indicators at different protocol or abstraction levels. Examples are interference level at the physical layer, delays at the MAC and network layers, and net data rate at session or application layers. The latest application layer indicators, also called key quality indicators (KQIs) [1], are especially relevant for the management of cellular networks. KQIs directly measure the service performance that the user experiences when executing particular services (e.g., web browsing, file download, video streaming [2], and video games [3]). A service provider can then use selected KQIs to improve its network operation and plan further expansions. Furthermore, when the final user of the system is a person, KQIs can be used to estimate subjective indicators, such as the mean opinion score (MOS) for voice services and PEVQ (perceptual evaluation of video quality) [4].

A third difficulty is implementing mechanisms that provide real-time assessment of the network status, potentially impacting the service provider’s OPEX and CAPEX. The scope of self-organized networks (SONs) [5] aims to address these demands.

Although indispensable for these three aspects, acquiring the KQIs implies facing considerable obstacles. Firstly, KQIs are out of the operator’s measurable scope, as they are part of the application layer. Although monitoring applications or deep packet inspection might be a solution to access such measures, the growing use of end-to-end high layer encryption and concerns about users’ privacy can make those approaches unsuitable for the task.

In this context, modeling KQIs from lower layer (accessible by the operator) measurements can be vital to estimate the application specific user performance. We look for computationally efficient algorithms capable of providing online results and suitable to be implemented in different network nodes, such as UEs, eNBs, and EPCs [6]. Simultaneously, the outputs of these algorithms are also time-series. The availability of time-indexed data is useful for additional post-processing by the operations and management (OAM) subsystem, where flexibility is a desirable feature to enable new choices of digital signal processing algorithms and the application of different machine learning (ML) techniques.

ML techniques are expected to play a relevant role in the management of wireless networks, as described by Ali et al. [7] and by Boutaba et al. [8]. Recent examples in the literature are given by Wang et al. [9], where the authors presented a robust architecture for artificial intelligence-enabled Internet of Things (AIoT) systems, and by Fiandrino et al. [10], which described an ML based general framework to optimize the operation of 5G and beyond networks. Machine learning mechanisms, such as recursive neural networks (RNNs), are also being applied to address other challenges in the context of cellular networks, as detalied by Wang et al. [11] on the security issue of voice cloning.

Narrowing the range of applications, one of the purposes of ML in wireless networks is the automation of service provisioning with a focus on the quality of service (QoS) and quality of experience (QoE), where KQIs provide relevant information for numerical processing by the ML algorithms. In this field, the work by Herrera-Garcia et al. [1] addressed the problem of KQI modeling using an ML approach based on regression techniques, but it did not consider an online, dynamic construction of the models. Baena et al. [12] extended the regression approach to consider the time dependency of KQI modeling in a video services scenario. However, the method requires prior knowledge of some parameters related to network configuration. Fan et al. [13] also proposed an ML based method to map KPIs to KQIs using sliding-window partitioning and random forest algorithms, but again without an explicit model for dynamic regression. Additional studies by Fortes et al. [14,15,16,17] have focused on the use of other high layer contextual information (e.g., location, social data) to support the management process in correlated yet different contexts (mainly SON), without explicit reference to multivariate time-series processing algorithms.

Going beyond the described state-of-the-art, the present paper defines a novel framework for estimating KQIs based on multivariate adaptive filters in conjunction with an ML clustering algorithm. The main distinction between this work and the ones mentioned above is twofold: First, it contains an explicit description of a time based approach suitable for online KQI modeling. This method has the flexibility to be used in conjunction with other ML techniques, and we provide suggestions of different algorithms that can be used within the general framework. The second distinction is the possibility to obtain distinct KQI models from measurements when facing a nonstationary environment or when there are different service configurations.

Adaptive filtering algorithms have suitable characteristics for dynamic KQI modeling, such as online training, flexibility to provide linear and non-linear mappings, the capability to handle multivariate time-series, and fast execution. Furthermore, it naturally provides an indicator of accurate modeling in a stationary environment, namely the estimation error obtained in the training phase. Proper handling of this estimation error in conjunction with a clustering algorithm is the basis of the framework, and to our best knowledge, it has not yet been applied to the problem of KQI modeling in wireless networks.

The rest of this paper is organized as follows: Section 2 provides the mathematical formalization for the problem and the notation used in the rest of the text. Section 3 presents the general description of the framework, while Section 4 details the algorithms used for KQI modeling in a non-stationary context. Section 5 shows (i) an initial assessment of the proposed approach using collected data from a real environment and (ii) open research challenges using the same general framework, but with different algorithms and slight variations. Finally, Section 6 summarizes the conclusions of this work.

## 2. Notation, Hypothesis, and Simplifications

Measurements gathered from wireless networks may be very different in nature and are originated from distinct network elements with different purposes. Here, we make one basic distinction between measurements, parameters, and auxiliary data.

Here, we call measurements what we get from the network. Typical representatives are:

- Data from the PHY/MAC protocol layers that can be obtained from the nodes. Typical examples are RSSI (received signal strength indicator) measured in eNB and drive tests logs.
- Classical KPIs from eNBs related to mobility and connection management, such as handover success rate and dropped call rates.

We call parameters data whose values:

- Can be set in equipment, such as: UE transmitter power; number of RF carriers; antenna downtilt;
- Numbers that characterizes a service: the minimum data rate for data services; maximum acceptable delay; file data size; network bandwidth. The popularization of software-defined radio (SDR) equipment can naturally blur the distinction between these two subgroups.

Finally, we call auxiliary data information that can be gathered from other sources than the wireless network, such as service type (video streaming, text messages, VoIP) and georeferenced information (demographic profiles, RF propagation environment).

In the rest of the text, we will use the notation defined in Table 1.

Using these definitions and notation, we can state our main working hypothesis:

**Hypothesis** **1.**  *Time stamps that come from different sources are consistent, i.e., the algorithms perform on the same sequence of time instants $\left[t_{1}\;\;t_{2}\;\;\ldots \;\;t_{n}\right]$ for all measurements.*

**Hypothesis** **2.**  *The set K can be obtained from M. In this front, some effort has been made to organize the set of measurement reports in standardization forums [18,19,20].*

## 3. Reference Model

**Figure 1:**
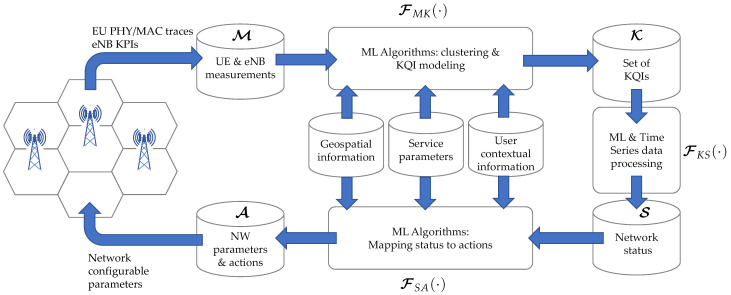
General information flow for self-organized network (SON) status prediction and assessment through key quality indicator (KQI) modeling and processing.

The proposed approach is based on the global architecture of Figure 1. From the measurements M obtained from the network, with the aid of information in auxiliary databanks, a mapping $F_{MK}\left(\cdot \right)$ is built to construct the set of KQIs K. Further processing can be used to obtain the maps $F_{KS}\left(\cdot \right)$ to obtain the network status S from the KQIs, as well $F_{SA}\left(\cdot \right)$, which maps S to the set of network parameters and actions A.

The present work focuses on the problem of building the map $F_{MK}\left(\cdot \right)$, using timestamped information originated from measurements at the physical layer, as well from service parameters.

Before going to the formal aspects, we briefly discuss qualitatively the main framework designed to obtain $F_{MK}\left(\cdot \right)$. The procedure can be divided into four steps: mapping, segmentation, clustering, and operation. Here, it is assumed that the set of training time-series is a good representation of the measurements (or input) space and that there is also a set of KQI time-series available for training, representative of the output space.

To produce the KQI mapping, we use a multichannel linear adaptive filter [21,22] with $d_{m}$ inputs, each one from a specific measurement $m_{n}^{1}$, $m_{n}^{2}$, …, $m_{n}^{d_{m}}$ collected at instant *n*. A selected ${\mathrm{KQI}}_{n}^{\mathrm{target}}$ is used to train the bank of filters, and the coefficients $w_{M,n}^{j}$, $j=1,2,\ldots ,d_{m}$, build the map $F_{MK}\left(\cdot \right)$.

It is worth mentioning that if the inputs are non-stationary, the adaptive filter will track the changes in the input space, and the set $\left\{w_{M}\right\}$ will convey only the latest representation of ${\mathrm{KQI}}^{\mathrm{target}}$ at the output space. If there is a need to cope with changes of regime, a mechanism for detecting them is needed. In such a case, a possible solution is to store the correspondent set of weights for each stationary section, as discussed further. This step is called  segmentation.

In parallel, the same input sequence $M_{n}$ is used to build a set of autoregressive models $w_{P,n}^{j}$, $j=1,2,\ldots ,d_{m}$. The purpose of this second filter bank is to provide a representation of the input space that can be used in the operation phase. Again, a set of $d_{m}$ adaptive predictors can be used to extract the parameters that represent the input space.

**Figure 2:**
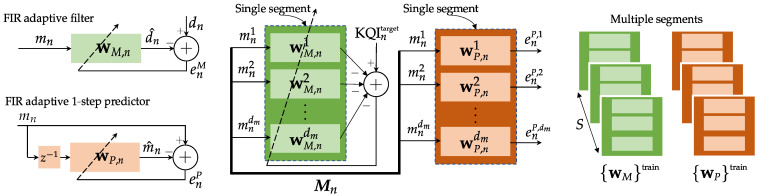
Adaptive filters used to construct representations of the measurement set $M_{n}$ (inputs) and output space (KQIs). Left: representation of a single adaptive filter with external training data $d_{n}$ (top), used as adaptive predictor (bottom); middle: multivariate adaptive filters (KQI mapping and multichannel predictors), for a single time-series segment; right: complete set of filter weights after the training phase.

At the end of this step, both sets of weights, generically referred to as ${\left\{w_{M}\right\}}^{\mathrm{train}}$ and ${\left\{w_{P}\right\}}^{\mathrm{train}}$, are stored to be used in the next phases. The full architecture is depicted in Figure 2.

The third phase, clustering, is aimed at extracting only the non-redundant models on the sets ${\left\{w_{M}\right\}}^{\mathrm{train}}$ and ${\left\{w_{P}\right\}}^{\mathrm{train}}$. This step is not strictly mandatory, but it was adopted as part of the framework with the objective of reducing the number of parameters needed to rebuild ${\mathrm{KQI}}_{n}^{\mathrm{target}}$ from the information available from the input space.

At the last step, the operation phase, new samples of the measurements are fed into a bank of predictors, using the same architectural structure used in the training step. Now, the system is constantly producing ${\left\{w_{P}\right\}}^{\mathrm{oper}}$, which can be compared with ${\left\{w_{P}\right\}}^{\mathrm{train}}$ to recover the correspondent ${\left\{w_{M}\right\}}^{\mathrm{train}}$. A criterion of the minimum distance between ${\left\{w_{M}\right\}}^{\mathrm{train}}$ and ${\left\{w_{M}\right\}}^{\mathrm{oper}}$ is used to select the best mapping in real time.

The idea behind the model is simple, and it can be used in conjunction with anomaly detection (ND) schemes [23,24,25,26] to identify changes in the input space in the non-stationary scenario. This can be done in both the training and operation phases. In the training phase, if there are significant alterations in the input space, new elements can be added to the ${\left\{w_{M}\right\}}^{\mathrm{train}}$ and ${\left\{w_{P}\right\}}^{\mathrm{train}}$ sets. Changes in $M_{n}$, such as a modification of a service parameter, will result in different weights due to the tracking nature of the adaptive filters.

These new elements can be labeled with an index that represents a quasi-stationary section of a general non-stationary behavior. The strategy is to build not only a single map, but a dictionary of maps (or an atlas) that provides the system a set of representations of the output space K as functions of the input space M, for different service or network conditions. In the operation phase, the ND mechanism can be used as a trigger to identify a new network operation regime not yet represented by ${\left\{w_{M}\right\}}^{\mathrm{train}}$ and request new maps to be added to the current atlas.

One possible approach to build new maps from the available data, in the training phase, is the utilization of the prediction errors:

(1) $$e_{n}^{P,j}=m_{n}^{j}-{\hat{m}}_{n}^{j}$$

$j=1,2,\ldots ,d_{m}$, where ${\hat{m}}_{n}^{j}$ is the estimation of the *j*th measurement at instant *n* made by the adaptive predictor. Analogously, the estimation error $e_{n}^{M}$ can also be used. By monitoring the values of $e_{n}^{P,j}$ and $e_{n}^{M}$, it is possible to make a decision about whether a new map is needed or not.

## 4. Proposed Algorithms

This section describes the specific algorithms developed to implement the KQI modeling functionality as defined in the previous section. These include the mechanisms associated with the determination of linear mappings, namely adaptive filers.

Adaptive filters make use of a recursive, sample based rule to update their parameters (or weights w). If the input environment is a stationary process, after a certain number of iterations, the weights will converge (in some statistical sense) to values regarded as optimum when they minimize a certain cost function J(w). Among different options for the algorithms, there are two common families of adaptive filters that provide solutions for the recursive problem of weight update, based on the formulation of J(w). The first is LMS based, derived from a statistical approach, where the steepest descent algorithm is widely used. The second is RLS based, where variations of the recursive least-squares algorithm are applied. The area of adaptive filtering is mature [27], and it was chosen due to: (i) simplicity of training, (ii) well-known properties of convergence and stability, and (iii) broad choice of options among existing algorithms.

### 4.1. Determination of Linear Mappings

The input of the multichannel adaptive filter responsible for providing $F_{MK}\left(\cdot \right)$ is a multivariate time-series with measurements $m_{n}^{j}$, $j=1,2,\ldots ,d_{m}$. For the sake of simplicity, we chose a finite impulse response (FIR) configuration with the same order *L* for each channel. A buffer of *L* past samples of each input channel *j* is then used to produce the correspondent $j^{th}$ output, as depicted in Figure 2. The output of all $d_{m}$ filters is now combined to produce ${\hat{\mathrm{KQI}}}_{n}^{\mathrm{target}}$:

(2) $${\hat{\mathrm{KQI}}}_{n}^{\mathrm{target}}=\sum_{j=1}^{d_{m}}m_{L,n}^{j}w_{M,n}^{j}.$$

The difference between the estimative and the selected ${\mathrm{KQI}}_{n}^{\mathrm{target}}$, the mapping error, is:

(3) $$e_{n}^{M}={\mathrm{KQI}}_{n}^{\mathrm{target}}-{\hat{\mathrm{KQI}}}_{n}^{\mathrm{target}}.$$

To provide processing speed and avoid numerical instability, we used the least-mean squares (LMS) algorithm to update the coefficients of each channel:

(4) $$w_{M,n+1}^{j}=w_{M,n}^{j}+2\mu \;e_{n}^{M}m_{L,n}^{j},$$

where μ is the step-size parameter of the LMS algorithm. Section 5.9 contains a brief discussion on different algorithms that may be used to update the coefficients. After convergence, the $F_{MK}\left(\cdot \right)$ mapping is stored as the set ${\left\{w_{M}\right\}}^{\mathrm{train}}$ of all coefficients $w_{M,n}^{j}$.

For the multichannel adaptive filter used to build the $j^{th}$ autoregressive model $w_{P,n}^{j}$ associated with the $j^{th}$ measurement, we calculated the corresponding prediction error $e_{n}^{P,j}$:

(5) $$e_{n}^{P,j}=m_{n}^{j}-m_{L,n-1}^{j}w_{P,n}^{j}.$$

We also used the LMS rule to update the regression coefficients for each channel *j*, $j=1,2,\ldots ,d_{m}$:

(6) $$w_{P,n+1}^{j}=w_{P,n}^{j}+2\mu \;e_{n}^{P,j}m_{L,n-1}^{j},$$

Again, all prediction filters have the same order *L*, and after convergence, the autoregressive coefficients constitutes the set ${\left\{w_{P}\right\}}^{\mathrm{train}}$. We also point out that there is no need to use the same order *L* for both mapping and prediction filters.

For the stationary case, both sets ${\left\{w_{M}\right\}}^{\mathrm{train}}$ and ${\left\{w_{P}\right\}}^{\mathrm{train}}$ will contain $d_{m}$ elements, each one of length *L*. Due to the tracking nature of adaptive filters, in a non-stationary environment, there is a need to identify changes in the input data that lead to a different $F_{MK}\left(\cdot \right)$. Therefore, a new time-series segment should be detected, and the corresponding mapping and predictor weights must be stored. Using *i* as the index for the segments, the weights for prediction and mapping, as well the prediction and estimation errors are expressed respectively as $w_{P,n}^{j,i}$, $w_{M,n}^{j,i}$, $e_{n}^{P,j,i}$, and $e_{n}^{M,i}$. If *S* is the number of segments, by the final training phase, there will be an atlas of *S* linear mappings for KQI estimation, as well as *S* banks of autoregressive models. A set of $S\cdot L\cdot d_{m}$ parameters will then represents $F_{MK}\left(\cdot \right)$.

### 4.2. Detection of Quasi-Stationary Segments

The detection of a new quasi-stationary state $s^{i+1}$ can be performed by different anomaly (or novelty) algorithms, such as PCA [28], convolutional neural networks [29], and the Kullback–Leibler divergence [25]. The proposed approach uses the available set of $d_{m}\times S$ prediction errors $e_{n}^{P,j,i}$ and a threshold $\gamma_{MAX}$ as follows: for all *i* segments, verify if:

(7) $$\gamma_{i}=\sum_{j=1}^{d_{m}}{∥e_{n}^{P,j,i}∥}^{2}\geq \gamma_{MAX}$$

is true. If $\gamma_{i}\geq \gamma_{MAX}$ for all segments, none of the previous *i* mappings is considered suitable, and a new segment is added. We justify the use of the prediction errors because a regime change in a single channel may lead to a different $F_{MK}\left(\cdot \right)$. Now, the corresponding coefficients $w_{M,n}^{j,i+1}$ and $w_{P,n}^{j,i+1}$ are updated according to Equations (4) and (6). No further modifications are made on any other filter coefficients, and the process continues while there is available training data.

Other criteria can be used to detect a new segment using the information available from the training, such as a weighted combination of $e_{n}^{P,j,i}$ (in *j* index) to favor selected measurements. Utilize the mapping error $e_{n}^{M,i}$ in conjunction with the prediction errors is also possible, but in our approach, only the prediction errors are available in the operating phase. Another option is to define a dissimilarity metric in the feature space, i.e., use the distance between ${\left\{w_{M,n}\right\}}^{\mathrm{train}}$ or ${\left\{w_{P,n}\right\}}^{\mathrm{train}}$ and their past versions at some previous instant n−T. Here, a threshold is also needed.

### 4.3. Clustering Phase

After segmentation, there is a total of S≥1 segments. Clustering can be performed on ${\left\{w_{P}^{i}\right\}}^{\mathrm{train}}$ or simultaneously in both sets ${\left\{w_{P}^{i}\right\}}^{\mathrm{train}}$ and ${\left\{w_{M}^{i}\right\}}^{\mathrm{train}}$ (i=1,2,…S). The latter option is preferred, as the clustering procedure should preserve the mappings $F_{MK}\left(\cdot \right)$. For instance, when using the self-organizing map (SOM) algorithm, its inputs would be:

(8) $$x_{in}^{i}=\left[\begin{array}{c} w_{M}^{i}\\ w_{P}^{i} \end{array}\right],$$

for i=1,2,…,S. For moderate values of *S*, e.g., S≤50, simpler methods of clustering can be performed, such as *k*-means.

### 4.4. Operation Phase

In this stage, new measurements are presented to the system. The mapping information is encoded in the sets ${\left\{w_{M}^{i}\right\}}^{\mathrm{train}}$ and ${\left\{w_{P}^{i}\right\}}^{\mathrm{train}}$. A prediction of the measurements is performed in this phase in the same fashion as in the training phase. The coefficients ${\left\{w_{P}\right\}}^{\mathrm{oper}}$ are compared with all ${\left\{w_{P}^{i}\right\}}^{\mathrm{train}}$, and the segment with the nearest distance is selected, i.e., choose the segment indexed by $s^{*}$ such that:

(9) $${∥w_{P}^{s^{*},\mathrm{train}}-w_{P}^{\mathrm{oper}}∥}^{2}$$

is minimum. Using the associated map coefficients $w_{M}^{s*,\mathrm{train}}$, the estimation of the KQI is produced. The complete procedure, implemented with the LMS algorithm, is presented as pseudocode in Algorithm 1.

The computational cost of the whole procedure and the memory requirements will strongly depend on the implementation choices. For instance, the number of arithmetic sums and multiplications for a typical LMS based algorithm is linear with the filter order *L*. Considering the multivariate approach with $d_{m}$ measurements, it remains linear, but now with order $L\times d_{m}$. The same general observation applies to the clustering algorithms, such as *k*-means, where the complexity depends on the number of clusters, the dimension of the input data set (here *L*), and the number of elements of the input data (here $S\times d_{m}$).
**Algorithm 1** KQI modeling using LMS multivariate adaptive filtering as pseudocode.**% Initialization**Define LMS parameter μ, the order *L* of adaptive filters, and threshold $\gamma_{MAX}$Assign small random values to all ${\left\{w_{M}^{i}\right\}}^{\mathrm{train}}$ and ${\left\{w_{P}^{i}\right\}}^{\mathrm{train}}$ coefficients**% Training Phase****for** Every time step *n* **do**    %Adaptive Filtering        Select the $i^{th}$ category to update the coefficients ${\left\{w_{M}^{i}\right\}}^{\mathrm{train}}$ and ${\left\{w_{P}^{i}\right\}}^{\mathrm{train}}$        Update mapping and prediction errors from Equations (3) and (5)        Update filter coefficients using Equations (4) and (6)    %Test for new stationary segment    **for** All *i* current segments **do**        Check if a new segment is needed using Equation (7)        **if** $\gamma_{i}\geq \gamma_{MAX}$ **then**           Add a new segment, and select it for weight update        **end if**    **end for****end for****% Clustering Phase**Perform simultaneous clustering on the ${\left\{w_{P}\right\}}^{\mathrm{train}}$ and ${\left\{w_{M}\right\}}^{\mathrm{train}}$ sets**% Operating Phase****for** Every time step *n* **do**    Update prediction filter coefficients ${\left\{w_{P}\right\}}^{\mathrm{oper}}$ using Equation (6)    Calculate the distances from ${\left\{w_{P}\right\}}^{\mathrm{oper}}$ to all entries in the clustered ${\left\{w_{P}\right\}}^{\mathrm{train}}$ set    Select index $s^{*}$ corresponding to the least distance according to Equation (9)    Use $s^{*}$ to recover the corresponding ${\left\{w_{M}^{s^{*}}\right\}}^{\mathrm{train}}$ mapping coefficients    Use Equation (2) to estimate the desired KQI**end for**

The next two sections show preliminary computational results with the purpose of assessing the framework concept. A systematic analysis, in different scenarios and with detailed statistical analysis of the results, is reserved for future investigations.

## 5. Concept Evaluation: Experiments, Results, and Discussion

The first set of computational experiments was conducted to assess the capability of the framework to model a chosen KQI from a single set of measurements. The data were based on the execution of the service file downloaded via FTP, and all experiments were conducted in the UMAHetNetnetwork [30]. The dataset, as described by Herrera-Garcia et al. [1], the details of which are further described throughout this section, provides a key example of KQIs at the application layer under variable configurations and radio situations. It also allows for a direct comparison with other regression mechanisms applied in that work.

**Figure 3:**
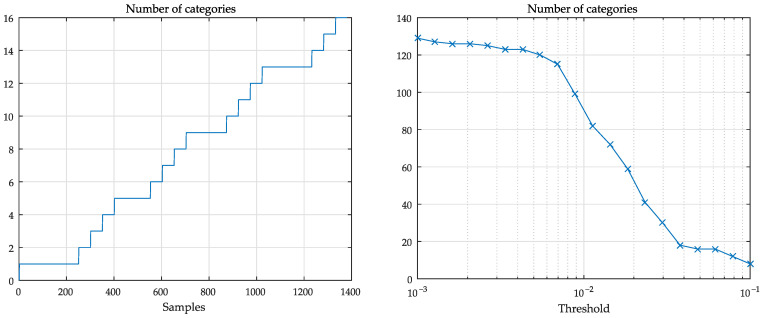
(**left**) evolution of the number of categories during the training phase. Compare with Figure 4. (**right**) typical behavior of the number of categories as a function of $\gamma_{MAX}$.

**Figure 4:**
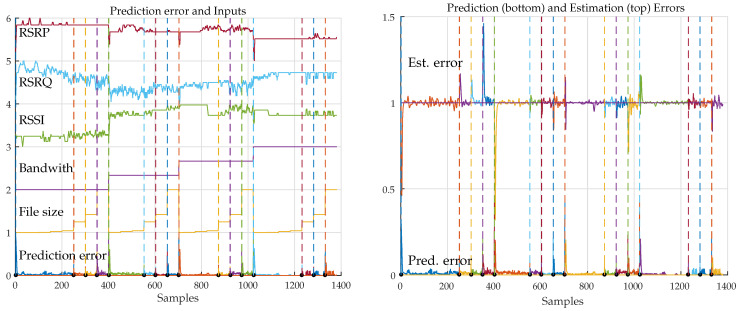
(**left**) input signals and sum of prediction errors (not in scale). (**right**) corresponding prediction and estimation errors. Dashed lines correspond to events with large prediction errors.

**Figure 5:**
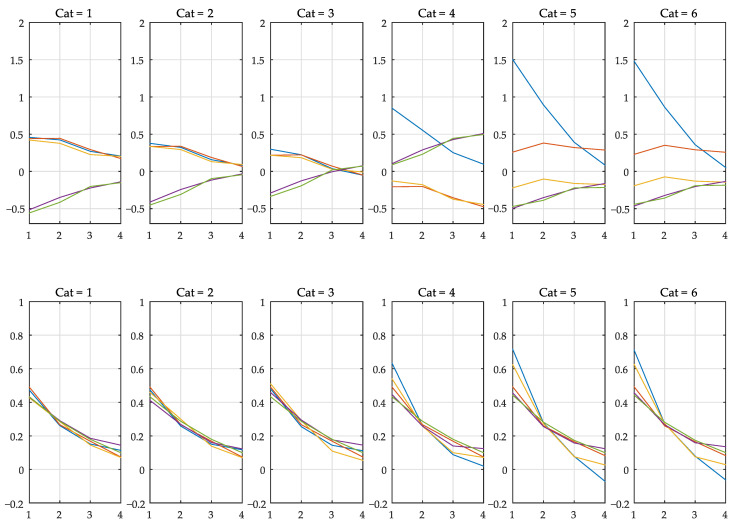
Map (first row) and prediction (second row) weights in the training phase, for the first six (from 16; see Figure 3) time-series segments. Each line in a graph represents the *j*-th group of coefficients associated with measurement $m_{n}^{j}$.

**Figure 6:**
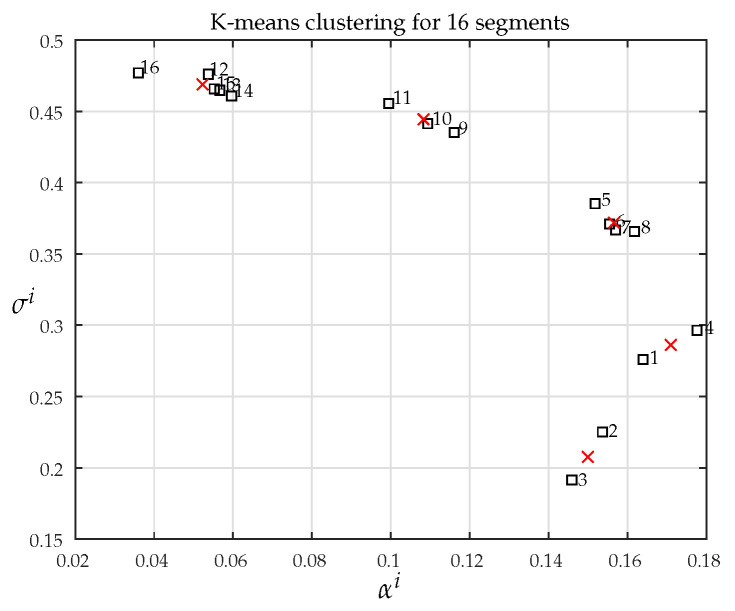
Result of *k*-means clustering with five categories. Cluster centers are indicated by red crosses.

**Figure 7:**
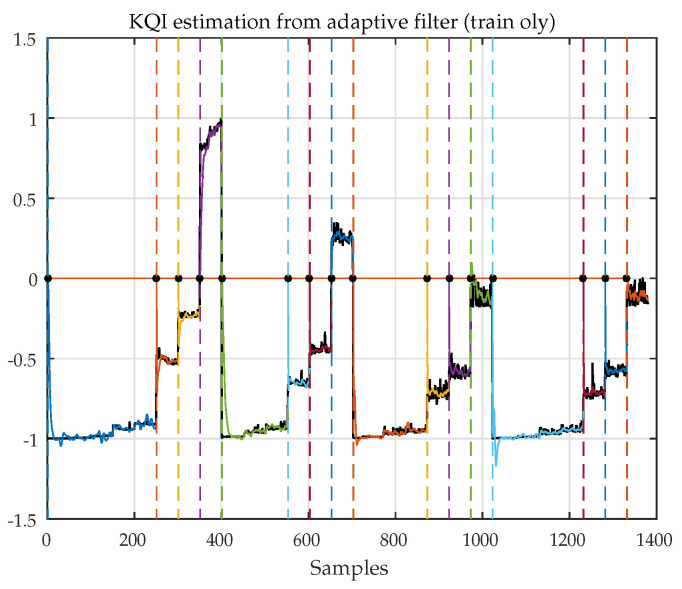
KQI “FTP session total time” as a function of RSSI, RSSQ, RSSP, bandwidth, and file size (colored by segment). Black: original data. Result obtained using the LMS based multivariate FIR adaptive filter.

**Figure 8:**
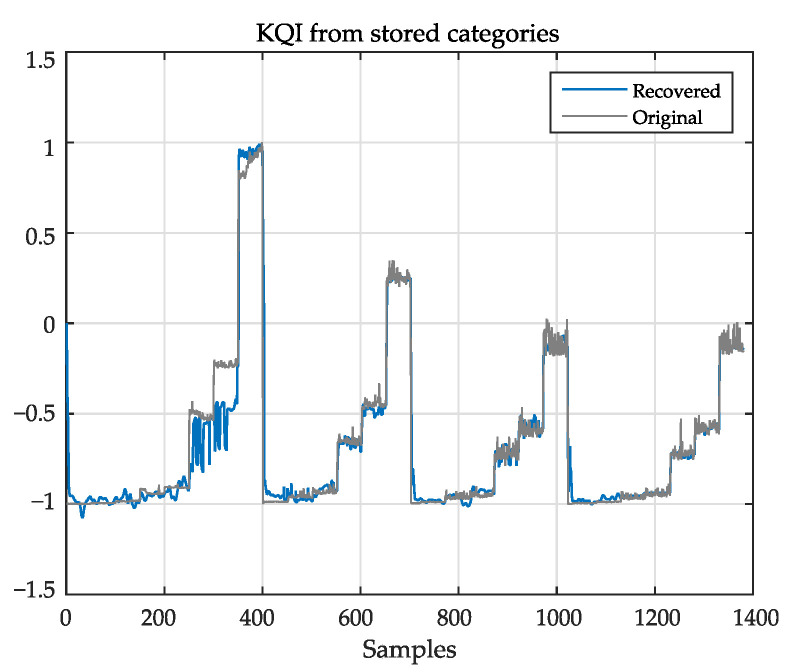
Blue: recovered KQI from ${\left\{w_{P}\right\}}^{\mathrm{oper}}$. Black: original data; blue: recovered KQI. Compare with Figure 7.

In this way, a single campaign dataset was used for training the system and to produce ${\left\{w_{M}^{i}\right\}}^{\mathrm{train}}$ and ${\left\{w_{P}^{i}\right\}}^{\mathrm{train}}$. The purpose of this setup is to validate the capability to perform segmentation and use the segments to recover the correct ${\left\{w_{M}^{i}\right\}}^{\mathrm{train}}$ from ${\left\{w_{P}^{i}\right\}}^{\mathrm{oper}}$. The same data are used in Figure 3, Figure 4, Figure 5, Figure 6, Figure 7 and Figure 8.

### 5.1. Measurement Dataset

Five measurements and three KQIs from the FTP service were used, as depicted in Table 2. Among these, three of them are related to RF MAC/PHY layer parameters, namely RSSI, RSRP (reference signal received power), and RSRQ (reference signal received quality), and two of them are parameters related to the provisioning of the FTP service: network bandwidth and file size. KQIs to be modeled are transmission rate, session total time, and session setup time.

### 5.2. Data Pre-Processing

Due to the large difference in the order of magnitude of the measurements and parameters, a normalization procedure was applied in order to mitigate numerical bias. All data were normalized to the range $\left[-1,1\right]$. It is worth mentioning that the final results may change if a different normalization procedure is applied or if another range is used. Furthermore, data from the lower layers are typically contaminated with noise and subjected to large variations due to the dynamic nature of the wireless channel. To this end, in these experiments, all PHY measurements (RSSI, RSRP, and RSRQ) were also filtered by a simple moving average filter with a window length of five samples.

### 5.3. Time-Series Segmentation and Prediction of Measurements

A graph of the number of segments, or categories, obtained in the training phase for the current example is depicted in Figure 3. The events that triggered the inclusion of a new segment were large prediction errors. In this case, the figure shows that the number of segments obtained at the end of the training phase was S=16. It is interesting to note that, in this particular case, new segments were added due the changes of measurements $m_{n}^{4}$ and $m_{n}^{5}$ (see Figure 4).

Figure 3 (right) also illustrates the typical dependence of the number of categories *S* according to the threshold $\gamma_{MAX}$, as a result of the procedure described in Section 4.2. As the number of categories is ultimately determined by the threshold $\gamma_{MAX}$, it is recommended to perform an intermediary step to optimize the threshold value. A brief analysis of this issue can be found in Section 5.9.

The prediction for $m_{n}^{j}$, j=1,2,…,5, was implemented using one-step ahead predictors of order L=4. Further adjustment of filter order and its effect on the final results are also expected to be performed in a further parameter optimization phase. As an example of the performance of the predictors, Figure 4 depicts, on the left, the five measurements and the sum of their correspondent prediction errors. At the bottom of the figure, it is possible to note the larger prediction errors $e_{n}^{P,j}$ at transitions of file size or bandwidth due to parameter changes. The graph on the right shows the same prediction errors at the bottom and, with a displacement of one unit in the y-axis, the correspondent mapping estimation errors $e_{n}^{M}$ for each category.

At the end of the procedure, a dictionary with all ${\left\{w_{P}\right\}}^{\mathrm{train}}$ and ${\left\{w_{M}\right\}}^{\mathrm{train}}$ is produced. As an example, Figure 5, gives the first six sets of weights (first row: ${\left\{w_{M}\right\}}^{\mathrm{train}}$; second row: ${\left\{w_{P}\right\}}^{\mathrm{train}}$). It is possible to notice, even visually, that segments 1 and 2 are similar, as well as Segments 5 and 6. This justifies the possible need for a clustering procedure on the set of filter coefficients at the end of the training phase. Alternatively, it is possible to use robust techniques with the objective of avoiding point anomalies that may unnecessarily increase the number of mappings.

### 5.4. Clustering

For this example, the number of segments is S=16. This is a relatively low number, and just for illustration purposes, we applied a *k*-means algorithm on the sets of ${\left\{w_{P}\right\}}^{\mathrm{train}}$ and ${\left\{w_{M}\right\}}^{\mathrm{train}}$. Now, the raw data used as input to the clustering algorithm must be associated with the joint set of weights, according to Equation (8).

As the filter order is *L* and the number of predictors is $d_{m}$, the elements of ${\left\{w_{P}\right\}}^{\mathrm{train}}$ and ${\left\{w_{M}\right\}}^{\mathrm{train}}$ were stored as $L\times d_{m}$ matrices. A first option would be to perform the clustering directly on the matrices, but here, we used a different method for visualization purposes. For each input vector $x_{in}^{i}$, we extracted two numbers: its mean $\alpha^{i}$ and its standard deviation $\sigma^{i}$.

(10) $$x_{in}^{i}=\left[\begin{array}{c} w_{M}^{i}\\ w_{P}^{i} \end{array}\right]\mapsto \left[\begin{array}{c} \alpha^{i}\\ \sigma^{i} \end{array}\right]$$

The pair of numbers ${\left[\alpha^{i}\;\;\sigma^{i}\right]}^{T}$ was then submitted to a *k*-means clustering algorithm with five classes. Results are depicted in Figure 6, where it is possible to notice the grouping of sequential indexes, in accordance with Figure 3.

At this stage, further analysis and improvements could be proposed, but have not been tested, such as performing the clustering taking into account the relative frequency of utilization of each segment.

### 5.5. Mapping and Estimation Errors

In Figure 7, it is possible to verify the performance of the LMS based multivariate adaptive filter that produces the set of coefficients ${\left\{w_{M}\right\}}^{\mathrm{train}}$. The chosen KQI is “session total time”, expressed as a linear combination of RSSI, RSSQ, RSSP, bandwidth, and file size. In this phase, it is expected that the estimation performs well, as long as the step-size parameter μ and the filter order are properly set. In the particular case studied here, the waveforms $m_{n}^{4}$ and $m_{n}^{5}$ have constant values, leading to potential numerical convergence problems if the filter order is too high and if stabilization procedures are not taken.

### 5.6. KQI Reconstruction from the Prediction Coefficients

Figure 8 shows the session total time KQI recovered from the prediction coefficients. In the operation phase, coefficients ${\left\{w_{P}\right\}}^{\mathrm{oper}}$ are compared with ${\left\{w_{P}\right\}}^{\mathrm{train}}$. The closest set of coefficients in ${\left\{w_{P}\right\}}^{\mathrm{train}}$ is selected, and the corresponding ${\left\{w_{M}\right\}}^{\mathrm{train}}$ are used to recover the KQI from the inputs. The reliability of the system depends on the consistent pairing of ${\left\{w_{P}\right\}}^{\mathrm{train}}$ and ${\left\{w_{M}\right\}}^{\mathrm{train}}$: if there are similar prediction weights associated with different mapping weights, the ambiguities in the operation phase will result in poor recovery.

### 5.7. Setup for Validation

For comparison purposes, from the same dataset used so far, $n_{MAX}=400$ samples were selected, now divided into disjoint training and testing subsets. As the predictor filters require a time window of length *L* from the input series to produce an estimate, the test samples cannot be randomly chosen as a single measurement $M_{n}$. With the objectives of (i) providing a preliminary assessment of the method and (ii) maintaining the sequential operation of the adaptive filters in the operation phase, one out of *p* samples from the dataset were taken sequentially through all measurements. This extraction results in two disjoint subsequences, both ordered in time, with lengths $n_{MAX}\cdot \left(p-1\right)p^{-1}$ and $n_{MAX}\cdot p^{-1}$. The first subsequence is used as the training set and the second as the testing set.

**Figure 9:**
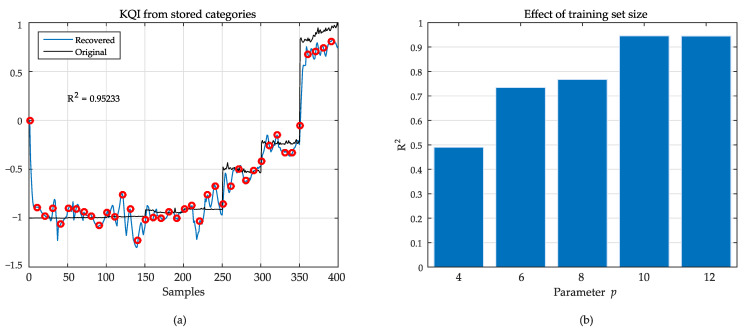
Recovered KQI from ${\left\{w_{P}\right\}}^{\mathrm{oper}}$ at left (**a**). Black: original data; blue: recovered KQI. Red dots are samples used for testing, not present in the training data. At right (**b**), the effect of the size of the training set in $R^{2}$.

Figure 9 depicts results using LMS filtering. In this example, we set p=10 to obtain the graph at the left, where it is possible to verify that the reconstructed signal follows the changes of the original KQI used for training. The coefficient of determination $R^{2}$ was used to evaluate the algorithm’s performance. This figure of merit measures how much the variance of a dependent variable (i.e., the estimated KQI) can be predicted from an independent one (i.e., the measured KQI). This is a widely extended measure to quantify the quality of regression mechanisms [31]. $R^{2}$ absolute values are defined in the range [0,1], where a value of one implies a perfect fit.

In this scenario, the approach provided a value of $R^{2}=0.95$, showing a very high performance. In the same Figure 9, on the right, it is possible to verify some degradation of the performance if there is a reduction of *p*. As smaller values of *p* reflect an increase in the proportion between testing and training sets, this behavior is expected.

### 5.8. Optimization of Free Parameters

Table 3 contains a list of parameters that can be optimized in the training phase. From them, we selected the threshold $\gamma_{MAX}$, directly related to the number of detected segments.

**Figure 10:**
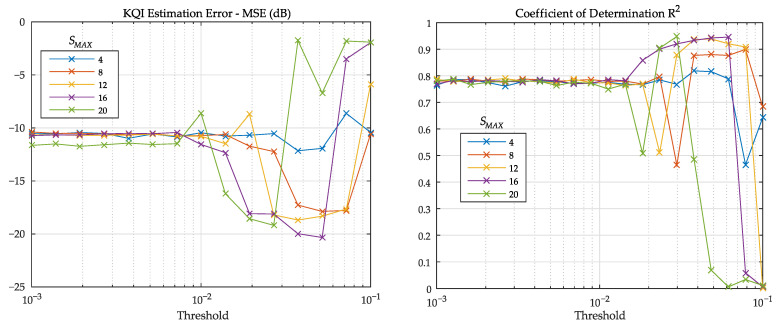
Estimation MSE (**left**) and coefficient of determination $R^{2}$ (**right**) as a function of prediction error threshold $\gamma_{MAX}$ using different values for the maximum number of segments $S_{MAX}$.

In this evaluation, the number of segments was limited to a maximum value $S_{\mathrm{MAX}}$, with five different values: 4,8,12,16,and20. For each value of $S_{\mathrm{MAX}}$, the threshold was changed from $10^{-3}\;\mathrm{to}\;10^{-1}$. The effect on the mean squared value of the estimation error:

(11) $$e_{n}^{\mathrm{est}}={\mathrm{KQI}}_{n}^{\mathrm{target}}-{\hat{\mathrm{KQI}}}_{n}^{\mathrm{target}}$$

is depicted in Figure 10.

Optimization of $\gamma_{MAX}$ is important to achieve good performance, but the other parameters from Table 3 also have an effect on $R^{2}$ (or equivalently, on MSE). In particular, the order of the FIR filters *L* cannot be made too large to avoid numerical instabilities. Furthermore, the LMS step μ has an optimal value that depends on the eigenvalues of the correlation matrix of the input data [32]. Results from Figure 10 were obtained with L=4 and μ=0.15, and similar outcomes were observed with simulations within the ranges of L∈(2,8) and μ∈(0.05,0.25).

Table 4 shows the results from this framework, labeled AMVTS (adaptive multivariate time-series), compared with other linear regression techniques using the $R^{2}$ parameter as a metric for comparison. We chose linear regression (LR), stepwise linear regression (SW-LR), and support vector regression (SVR), as detailed by Herrera-Garcia et al. [1], where the authors used the same dataset. As can be seen, AMVTS obtains values up to 0.95 for “transmission rate”, improving the good performance of SVR for this KQI by 6%. Moreover, AMVTS achieves the same performance with the best “session setup time” KQI technique, where the performance was 0.57. AMVTS performed worse only with the KQI “session total time”, where all linear techniques had poor results due to the fact that these KQIs’ values were nearly constant and completely dependent on the computational speed of the FTP server.

### 5.9. Discussion and Alternative Approaches

This section identifies and discusses the open research lines generated by the present study, establishing a roadmap for future works and improvements. These focus on three three lines: variations of the general framework, application of the framework to the SON context, and further research.

#### 5.9.1. Variations on the General Framework

In the presented framework, the time-series segmentation treats service parameters as components of the multivariate time-series. If the number of combinations of the service parameters is discrete and relatively small, one alternative is to keep all measurements and KPIs as the only signals to be used as the inputs of the adaptive filters and use the information in the service parameters as pointers to specific subsets of $w_{P,n}^{j,i}$ and $w_{M,n}^{j,i}$. Clustering can then be performed to identify which group of parameters leads to similar representations.

As an example, in the specific case reported (refer to Table 2), only $m_{n}^{1},\;m_{n}^{2},\;m_{n}^{3}$ would be utilized to produce local approximations of $F_{MK}\left(\cdot \right)$, and the 8×4=32 possible combinations of $m_{n}^{4}$ and $m_{n}^{5}$ would define 32 sets of $w_{P,n}^{j,i}$ and $w_{M,n}^{j,i}$.

Another interesting possibility is the utilization of the reverse mapping $F_{MK}^{-1}\left(\cdot \right)$, using the KQIs as the input time-series and the measurements as the outputs. A content service provider (SP), such as a streaming video service, may need to know if the wireless network can provide a pre-defined level of quality. In this scenario, the SP would infer the KPIs and the PHY/MAC measurements’ values from the KQIs and adjust all service configurations accordingly.

The proposed KQI modeling can be modified by changing its main algorithms, but maintaining the general framework. Some suggestions are:

- Choice of adaptive filter algorithms: Different options for LMS and recursive least squares (RLS) families can be found in Diniz [33], Sayed [34] and Haykin [32]. It is also possible to use a non-linear adaptive filtering approach, such as radial basis functions (RBFs), multilayer perceptrons (MLPs), and Volterra filters. The FIR structure can also be changed to IIR (Infinite-Implulse Response) as detailed by Regalia in [35], order-recursive lattice filters, or a stated based approach using Kalman filtering and its variations as described by Haykin in [36].
- Detection of quasi-stationary segments: A suggestion to extend the proposed method is implementing prediction filters that compute not only samples one step ahead, but also *p* steps ahead. A vector of *p* prediction errors can be used to provide a more reliable decision on creating a new category.
- Clustering strategies: A possibility beyond the minimization of redundancy between different time segments is to perform the clustering, not within a segment, but among the measurements $m^{j}$, to reduce the input dimensionality $d_{m}$.
- Different options to build representations of M and K spaces. The proposed method builds the feature representation of measurements and KQIs via adaptive filter coefficients. These are not the only option, and the efficiency of other indirect features can be investigated. Immediate suggestions based on the speed of calculations are spectral analysis via FFT (with a link to segmentation as pointed by Perron in [37]) and wavelets. The choice of the best feature space is this multivariate time-series problem is not obvious, and a deeper look at the subject may be worth the effort.

#### 5.9.2. Application of the Framework to the SON Context

Self-healing (SH) techniques can make use of the proposed algorithms. The network “homeostasis” is provided through a series of self-regulation mechanisms through closed-loop mechanisms, following the same general schematic of Figure 1.

A first impression of the direct applicability of the proposed framework for SH purposes is the use of $F_{KS}\left(\cdot \right)$, to monitor the system status in the eNB scope. Once the KQI time-series is obtained, the same adaptive prediction and mapping approach can produce a time-series $s_{n}$ representing the network status. After proper identification of common faulty states, self-recovering procedures can then use these states to circumscribe a faulty node’s effects in its neighborhood. The monitoring of the current status and prediction of its future values also allows the change of posture, from a reactive standpoint reacting to a failure or misconfiguration, to a functional perspective seeking automated preventive actions.

Closing the loop via $F_{SA}\left(\cdot \right)$ is challenging due to several reasons:

- The temporal granularity of the sets S and A can be very different, and the information loopback cannot be unstable. This reinforces the need for reliable maps obtained from $F_{MK}\left(\cdot \right)$ and $F_{KS}\left(\cdot \right)$.
- The actions should work in harmony with collaborative techniques, such as coordinated multipoint (CoMP), ICIC (inter-cell interference cancellation), and network coding.
- One single status $s_{n}$ may be related to a set of actions in A. In this scenario, the time horizon of observation of $s_{n}$ must be properly tuned such that a specific sequence of states is properly mapped to their correspondent sequences in A.
- The action performed in a specific cell may affect its neighboring cells. One interesting technique that could be assessed to cope with the spatial nature of effects is adaptive diffusion mechanisms [38], which optimize cost functions over a network of nodes.
- Typical networks are deployed in a multi-vendor environment, and node parameters are usually vendor-specific. Furthermore, the management information base (MIB) of network nodes may have hundreds of parameters. In this scenario, full automation can be costly to implement and face resistance from the service providers.

#### 5.9.3. Further Research

Two topics still need a detailed assessment of their technical feasibility. The first is focused on the measurements and database building via a surplus of computing power at network nodes to provide better KPIs and KQIs. Some $F_{MK}\left(\cdot \right)$ maps can be built as background processes called by the operating system at the network mobile nodes. This decentralized approach requires ML algorithms and auxiliary databases in Figure 1 to be, if not entirely, at least partially distributed. The OAM wireless system could be responsible for implementing other intensive data processing layers to cope with the complex interrelationships of information, such as spatial interdependence and user profiles. Related to this front line, another interesting possibility is to use the Age of Information concept [39] to relax the implicit constraints present in the recursive weight update of the multichannel adaptive filters.

A second topic is the investigation of new mappings from KQI to quality of experience (QoE). As quality is inherently service-dependent and possesses a subjective nature, QoE models are built typically for a specific traffic type, such as the ITUrecommendations for video services [40]. If Q is a comprehensive set or a representative indicator of the user’s final experience, a suggestion would be the search for new mappings $F_{KQ}\left(\cdot \right)$ from KQIs K to Q using the same adaptive modeling and segmentation strategies adopted to build $F_{MK}\left(\cdot \right)$.

## 6. Conclusions

The proposed method can produce KQI estimates from network measurements, with a performance comparable to other linear regression models. Its general framework allows flexibility to build the measurement-to-KQI maps by utilizing other choices than LMS based adaptive FIR filters, such as RLS based ones or non-linear adaptive filtering. In a nonstationary environment, the continuous generation of new KQI models is possible, but as the size of the atlas required to represent different mappings increases, we recommend using clustering techniques to reduce the number of parameters. Its applicability to the SON context is also possible, where the proper handling of the measurements timestamps could be adopted under the lens of the Age of Information paradigm [41], integrating the concept of the freshness of the data in the construction of the models and utilizing a restricted set of actions to be performed at the nodes before closing the information loop.

## Figures and Tables

**Table 1:** Mathematical notation: main symbols with short descriptions.

Symbol	Meaning
M,K,S,A	general sets of (respectively): data measurements, KQIs, network status and actions
$F_{XY}\left(\cdot \right)$	mapping between sets X and Y
n,j,i	indexes used, respectively, for: time instant, input data channel, and time-series segment
$M_{n}$	multivariate measurements gathered at time *n*, dimensions $d_{m}\times 1$
$m_{n}^{j}$	$j^{th}$ (from $d_{m}$) measurement gathered at instant *n*
$m_{L,n}^{j}$	$j^{th}$ measurement time-series window from time n−L+1 up to *n*, dimensions 1×L
${\mathrm{KQI}}_{n}^{\mathrm{target}}$	selected KQI to be modeled, at time *n*
$\left\{w_{M}\right\}$	generic weights of adaptive filters
$w_{M,n}^{j}$	$j^{th}$ (mapping) adaptive filter weights at time *n*, dimensions L×1
$w_{P,n}^{j}$	$j^{th}$ (prediction) adaptive filter weights at time *n*, dimensions L×1
$e_{n}^{P,j}$	$j^{th}$ prediction error at time *n*
$e_{n}^{M}$	mapping estimation error at time *n*

**Table 2:** Dataset used in the experiments from UMAHetNet. RSRP, reference signal received power; RSRQ, reference signal received quality.

Signal	Type	Variable
RSSI	RF PHY	$m_{n}^{1}$
RSRP	RF PHY	$m_{n}^{2}$
RSRQ	RF PHY	$m_{n}^{3}$
Network bandwidth	Parameter (4 values)	$m_{n}^{4}$
File size	Parameter (8 values)	$m_{n}^{5}$
Transmission rate	FTP KQI	$k_{n}^{1}$
Session setup time	FTP KQI	$k_{n}^{2}$
Session total time	FTP KQI	$k_{n}^{3}$

**Table 3:** Free parameters to be optimized.

Parameter	Meaning	Algorithm
μ	LMS step size	LMS
*L*	Order of FIR filters	LMS
$\gamma_{MAX}$	Prediction error threshold	Segmentation

**Table 4:** Comparison with other linear regression techniques using the $R^{2}$ performance metric (results from Herrera-Garcia et al. [1]). AMVTS, adaptive multivariate time-series; SW-LR, stepwise linear regression.

KQI	AMVTS	LR	SW-LR	SVR
Transmission rate	0.95	0.66	0.89	0.82
Session setup time	0.57	0.14	0.34	0.57
Session total time	0.03	0.03	0.05	−0.01

## Data Availability

Not applicable.
